# Effective filtering strategies to improve data quality from population-based whole exome sequencing studies

**DOI:** 10.1186/1471-2105-15-125

**Published:** 2014-05-02

**Authors:** Andrew R Carson, Erin N Smith, Hiroko Matsui, Sigrid K Brækkan, Kristen Jepsen, John-Bjarne Hansen, Kelly A Frazer

**Affiliations:** 1 Department of Pediatrics and Rady Children’s Hospital, University of California San Diego, San Diego, USA; 2 Department of Clinical Medicine, Hematological Research Group, University of Tromsø, Tromsø, Norway; 3 Division of Internal Medicine, University Hospital of North Norway, Tromsø, Norway; 4 Clinical and Translational Research Institute, University of California, San Diego, USA; 5 Department of Clinical Medicine, University of Tromsø, Tromsø, Norway; 6 Moores UCSD Cancer Center, University of California San Diego, La Jolla, CA, USA

**Keywords:** Next generation sequencing, Single nucleotide variants, Genotyping, Imputation, Genomics

## Abstract

**Background:**

Genotypes generated in next generation sequencing studies contain errors which can significantly impact the power to detect signals in common and rare variant association tests. These genotyping errors are not explicitly filtered by the standard GATK Variant Quality Score Recalibration (VQSR) tool and thus remain a source of errors in whole exome sequencing (WES) projects that follow GATK’s recommended best practices. Therefore, additional data filtering methods are required to effectively remove these errors before performing association analyses with complex phenotypes. Here we empirically derive thresholds for genotype and variant filters that, when used in conjunction with the VQSR tool, achieve higher data quality than when using VQSR alone.

**Results:**

The detailed filtering strategies improve the concordance of sequenced genotypes with array genotypes from 99.33% to 99.77%; improve the percent of discordant genotypes removed from 10.5% to 69.5%; and improve the Ti/Tv ratio from 2.63 to 2.75. We also demonstrate that managing batch effects by separating samples based on different target capture and sequencing chemistry protocols results in a final data set containing 40.9% more high-quality variants. In addition, imputation is an important component of WES studies and is used to estimate common variant genotypes to generate additional markers for association analyses. As such, we demonstrate filtering methods for imputed data that improve genotype concordance from 79.3% to 99.8% while removing 99.5% of discordant genotypes.

**Conclusions:**

The described filtering methods are advantageous for large population-based WES studies designed to identify common and rare variation associated with complex diseases. Compared to data processed through standard practices, these strategies result in substantially higher quality data for common and rare association analyses.

## Background

Whole exome sequencing (WES) is rapidly becoming the preferred method of analysis to study the genetic basis of disease in large cohorts of patient and control samples. WES studies examine the roles of both rare and common variants and, thus, have a distinct advantage over array-based technologies which generally focus on common variants. While common variants typically have modest effect sizes, rare variants, especially those in coding regions, can have larger effect sizes with greater potential to influence disease [1-6]. WES has been successfully utilized in numerous studies to identify functional mutations in Mendelian and rare diseases as well as cancer, where small numbers of variants with large effects sizes are expected to be the major contributors to the disease [7-16]. In contrast to these disorders, where few samples may be sufficient to reveal causative mutations, the detection of associated variants in complex disorders requires larger cohorts to adequately detect associations in common variants with weak effects sizes and to identify sufficient numbers of rare variants to achieve adequate power to detect association using burden and collapsing methods [17-19].

While WES sequencing studies have many advantages over array-based analyses, they are also susceptible to higher levels of genotyping errors [20-23]. These errors are generated throughout the sequencing process, especially at sites with low coverage or variants with low minor allele frequency (MAF). While population-based variant callers, such as the Genome Analysis Toolkit (GATK) [24], have improved the accuracy of genotypes for low frequency variants, they perform poorly when identifying singletons and doubletons [25]. Therefore, rare variants have a high heterozygote to homozygote error rate. Alternatively, as the MAF increases, homozygote to heterozygote errors increase in likelihood.

Genotype errors affect both common variant (single marker) association tests as well as rare variants collapsing association methods [26]. Non-differential errors (with equal error rates in cases and controls) generally don’t affect type I errors in association analyses, but they do significantly decrease statistical power [25]. In fact, heterozygote to homozygote errors markedly decrease power, with the minimum sample size required to observe statistical significance increasing to infinity as the MAF of the variant drops to zero [27,28]. Thus, rare variant association tests, which collapse genotypes from multiple variants with very low MAFs into single markers, are particularly sensitive to this type of genotyping error. Therefore, applying stringent filtering methods to improve the accuracy of genotypes and variants is essential for achieving the variant calling accuracy in large WES datasets required to precisely detect signals in rare variant collapsing association tests [25,26].

Software suites, such as the GATK [24], have been designed to manage large-scale sequencing projects. GATK’s best practices includes a variant filtering step following Variant Quality Score Recalibration (VQSR). This “VQSR filter” uses annotation metrics, such as quality by depth, mapping quality, variant position within reads and strand bias, from “true” variants (variants found in HapMap phase 3 release 3) to generate an adaptive error model. It then applies this model to the remaining variants to calculate a probability that each variant is real. Using this recalibrated quality score, users can filter lower quality variants. GATK recommends choosing a threshold that maintains 99% sensitivity for the “true” variants. However, recent studies have shown that unvalidated variants remain in datasets after following GATK’s best practices including VQSR and filtration [29]. In addition, the VQSR filter does not explicitly filter genotypes, allowing low quality genotypes generated at variant sites that pass the VQSR filter to persist in the VQSR filtered dataset. These low quality genotypes are a major source of errors in sequencing studies, significantly lowering the power in downstream association analyses. Lastly, GATK also notes that VQSR works best for WES with a minimum of 30 samples, indicating a need for appropriate thresholds that can function outside of VQSR. Overall, GATK filtering has limitations; GATK documentation itself recommends the implementation of additional dataset specific filters after VQSR filtration.

Along with sequenced variants, recent WES studies [30,31] have employed imputation methods to calculate the genotypes of common variants to use as additional markers in association analyses. Importantly, imputation expands the investigation beyond the exome and allows for the identification of quantitative trait loci within adjacent non-coding enhancer and other regulatory sequences which are known to harbor important variants influencing disease [32]. However, these imputation methods can generate inaccurate genotypes [33,34]. Again, these genotype errors decrease the statistical power to detect associations with complex disorders [35]. To date, no standard filtering methods have been established for genotypes imputed from WES data.

Here we describe effective data filtering methods that, when implemented between the GATK variant calling and VQSR filtering steps, improve the sequenced and imputed single nucleotide variant (SNV) quality in large-scale WES genetic studies. We focus on showing improvements compared to GATK’s Best Practices because a recent publication has shown that GATK is the best variant caller for general NGS analyses [36]. While filtering to improve the quality of insertion and deletion (indel) variants is also important, here we focus only on SNVs. We evaluate VQSR and prospective novel filters by calculating the non-reference concordance with an alternate dataset generated by genotyping 10 individuals using the Illumina HumanExome BeadChip, which contains >240,000 predominantly exonic markers. We also evaluated the ratio of transitions to transversions (Ti/Tv) in the identified SNVs. While Ti/Tv ratios are only an approximate measure of quality, higher Ti/Tv ratios are associated with lower false positives, with high quality exome variant datasets expecting to have Ti/Tv ratios between 2.8 and 3.0 [37-39]. We established filtering criteria by investigating quality metrics at both the genotype and variant levels. GATK variant calling generates genotype-level quality metrics including depth of data (DP) and genotype quality (GQ). DP values represent the number of reads passing quality control used to calculate the genotype at a specific site in a specific sample, with higher values for DP generally leading to more accurate genotype calls. GQ is a Phred-scaled value representing the confidence that the called genotype is the true genotype. Again, higher values reflect more accurate genotype calls.

In addition to improving the genotype qualities, we hypothesized that further variant filtration could improve the quality of the variants dataset. While VQSR uses various annotation values, including quality by depth, mapping quality, variant position within reads and strand bias, to recalibrate the quality score before filtering, it does not use Hardy-Weinberg equilibrium (HWE), average genotype quality or “call rate” (the % of samples with a non-missing genotype) to filter out low quality variants. HWE, quality, and call rate, are common metrics used for filtering variants from genotyping arrays. As such, establishing thresholds for these variant metrics may have corresponding utility in sequencing studies.

Due to the rapid development of sample preparation and sequencing technologies, large WES studies often generate data in sample batches using different versions of target capture and/or sequencing reagents. This creates data heterogeneity among the samples due to differences in sequencing coverage and can result in distinct variant qualities and call rates between batches. Thus, we investigated the importance of separating WES samples into batches and determined that this is a critical step to perform prior to filtering in order to achieve the highest quality variant dataset.

These methods appreciably improve data quality, compared to data filtered on VQSR alone, by removing more discordant genotypes, leading to a higher non-reference genotype concordance, and improving the Ti/Tv ratio. Application of these filters results in a significantly improved large-scale WES dataset. By removing non-differential errors, these filters theoretically increase the power to identify rare variants [25] underlying the genetic basis of complex diseases.

## Results

### Exome sequencing, variant calling and standard GATK VQSR filtering

As part of a large case-control study, we sequenced the exomes of 920 samples from a Norwegian population to an average depth of 100× in target regions, with an average of 82.5% of the target base pairs having at least 30× coverage. Using GATK best practices v3 [24] we identified 573,074 SNVs (356,932 known, matching a variant in dbSNP Build 135, and 216,142 novel) with 404,907,261 genotypes (including 362,659,468 homozygous reference and 42,247,793 non-reference gentoypes; the average variant has 707 samples with a non-missing genotype) in the 920 samples. Following VQSR filtering, 494,688 SNVs (323,791 known and 170,897 novel) and 352,729,725 genotypes (318,551,885 homozygous reference and 34,177,840 non-reference genotypes; the average variant has 713 samples with a non-missing genotype) were retained.

### Quality of the unfiltered and VQSR filtered datasets

To assess the accuracy of the genotype calls, we genotyped 10 of the 920 samples using Illumina HumanExome BeadChips, which assay >240,000 predominantly exonic markers. From these, only high quality HumanExome array genotypes passing a stringent filter (GCScore ≥ 0.3) were considered. This resulted in 2,384,928 genotypes with an average SNP call rate of 98.8% per sample. Of these genotypes, 696,604 genotypes could be compared with a corresponding genotype from the unfiltered WES dataset (Additional file 1).

**Figure 1:**
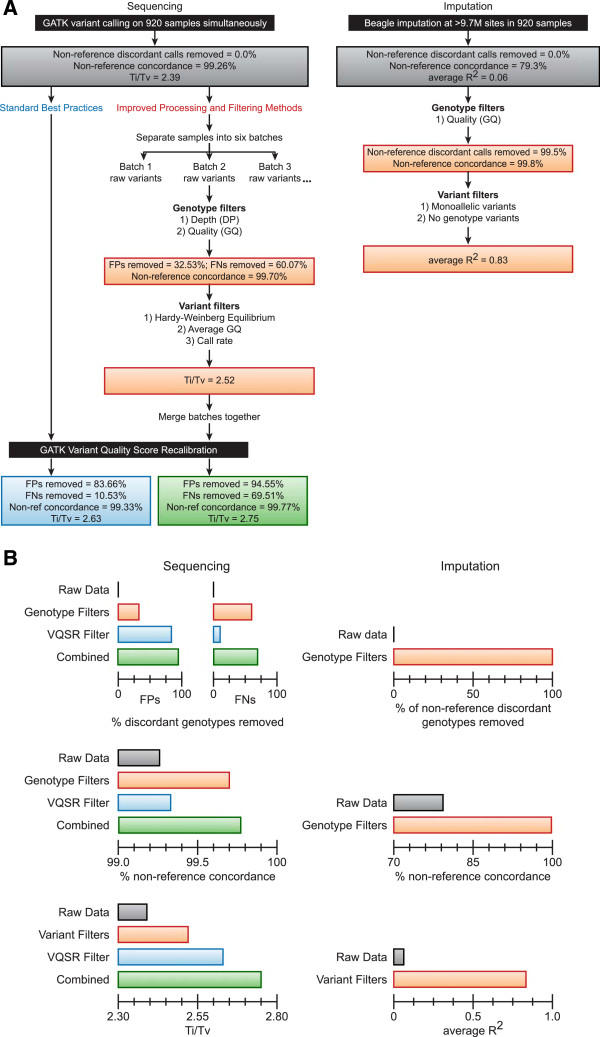
**Summary of methods and improved data quality from genotype and variant filters. A)** Left panel illustrates the standard filtering method (left side) compared to the proposed genotype and variant filtering method (right side) for sequencing data. Right panel illustrates the method used for genotype and variant filtering of imputed data. The quality metrics resulting from standard filtering (blue box), proposed genotype and variant filters (orange boxes), and a combination of these methods (green box) are compared to the quality of the unfiltered data (grey boxes). **B)** Quantitative comparisons of quality improvement are depicted for both sequencing and imputation filters at both genotype (% of discordant genotypes removed and % concordance) and variant (Ti/Tv and R^2^) levels. Box colors match the boxes in **A)**.

We calculated the genotype concordance between the sequencing calls and the exome array, where concordance is defined as the percent of identical, or concordant, genotypes out of the total number of compared genotypes. To avoid artificially bolstering concordance by including homozygous reference matches, we calculated concordance separately for exome array homozygous reference genotypes (n = 622,516) and exome array non-reference (heterozygous and homozygous alternate) genotypes (n = 74,088) (Additional file 1). Before applying any filters to the WES dataset, the genotype concordance with exome array non-reference genotypes was 99.26%. After applying the VQSR filter, 99.33% of the remaining genotypes were concordant (Table 1 and Additional file 1). Since the VQSR filter identifies high quality variant loci, but does not target specific genotypes, low quality genotypes remain in the WES dataset. For example, 11,453,170 low depth genotypes (DP < 8) and 11,733,096 low quality genotypes (GQ < 20, corresponding to a >1% likelihood of being an incorrect genotype call) remain in the dataset after VQSR filtering (Additional file 2). Overall, the VQSR filter removed 10.53% of the genotypes that were discordant with the non-reference exome array genotypes while removing 0.64% of the non-reference concordant genotypes (Figure 1, Table 1 and Additional file 1).

**Table 1:** Genotype concordance between WES genotypes and exome array genotypes in 10 samples

**Filters**		**Number of concordant genotypes**	**Number of discordant genotypes**^**†**^	**Concordance**	**% of concordant genotypes removed**	**% of discordant genotypes removed**
**Genotype**	**Variant**					
None	None	73,537 (621,855)	551 (661)	99.26% (99.89%)	NA	NA
None	VQSR	73,069 (608,065)	493 (108)	99.33% (99.98%)	0.64% (2.22%)	10.53% (83.66%)
DP	None	72,104 (611,606)	236 (627)	99.67% (99.90%)	1.95% (1.65%)	57.17% (5.14%)
GQ	None	72,689 (610,682)	225 (460)	99.69% (99.92%)	1.15% (1.80%)	59.17% (30.41%)
DP & GQ	None	71,986 (610,108)	220 (446)	99.70% (99.93%)	2.11% (1.89%)	60.07% (32.53%)
DP & GQ	VQSR	71,552 (597,070)	168 (36)	99.77% (99.99%)	2.70% (3.99%)	69.51% (94.55%)

Non-reference genotypes are shown above reference genotypes in brackets.

Non-reference genotypes include genotypes that are heterozygous or homozygous alternate in the exome array. Reference genotypes include genotypes that are homozygous reference in the exome array.

^†^ Includes heterozygote to homozygous alternate mismatches.

(See Additional file 1).

In addition to genotype concordance, we also calculated the sensitivity and specificity of the WES genotyping using the exome array genotypes as the “gold standard”. For this, we define true negatives (TN) as identical homozygous reference genotype matches and true positives (TP) as identical heterozygous or homozygous alternate genotype matches. False negatives (FN) are instances where the WES data is missing at least one alternate allele, while false positives (FP) are instances where the WES data has at least one extra alternate allele (Additional file 3). From this calculation we observe a sensitivity and specificity of 99.26% and 99.89%, respectively, in the unfiltered WES dataset. These values improve to 99.33% and 99.98% after the VQSR filter is applied (Table 2 and Additional file 3).

**Table 2:** Sensitivity and specificity of WES genotypes for exome array genotypes in 10 samples

**Filters**		**TP**^**1**^	**TN**^**2**^	**FP**^**3**^	**FN**^**4**^	**Sensitivity TP/(TP+FN)**	**Specificity TN/(TN+FP)**
**Genotype**	**Variant**						
None	None	73,537	621,855	661	551	99.26%	99.89%
None	VQSR	73,069	608,065	108	493	99.33%	99.98%
DP	None	72,104	611,606	627	236	99.67%	99.90%
GQ	None	72,689	610,682	460	225	99.69%	99.92%
DP & GQ	None	71,986	610,108	446	220	99.70%	99.93%
DP & GQ	VQSR	71,552	597,070	36	168	99.77%	99.99%

^1^TP = exact match of non-reference genotype; Ref/Alt with Ref/Alt or Alt/Alt with Alt/Alt.

^2^TN = exact match of reference genotype; Ref/Ref with Ref/Ref.

^3^FP = additional alternate allele in WES genotype; Ref/Ref with Ref/Alt or Ref/Ref with Alt/Alt or Ref/Alt with Alt/Alt.

^4^FN = missing alternate allele in WES genotype; Ref/Alt with Ref/Ref or Alt/Alt with Ref/Ref or Alt/Alt with Ref/Alt.

(See Additional file 3).

To further evaluate the variant quality of these datasets, we measured their Ti/Tv ratios. The unfiltered variant dataset has a Ti/Tv of 2.25. After applying the VQSR filter, the Ti/Tv ratio improved to 2.53 (Figure 1). While this is a significant improvement from the unfiltered dataset, the Ti/Tv ratio of the VQSR filtered variants is still below the expected ratio of 2.8 for high quality datasets. Based on these quality measurements, we posited that implementing additional filtering methods in conjunction with the standard VQSR filter would further improve the quality of the final variant dataset at both the genotype and variant levels.

### Separating samples into batches prior to filtering

During the course of our research, we incorporated technology improvements into our study design despite knowing that different clustering and targeting protocols would lead to batch effects caused by differences in factors such as target coverage (Additional file 4). During our study, the Illumina TruSeq PE Cluster Kit improved from version 2 (93 samples) to version 3 (827 samples), and the Agilent SureSelect target enrichment improved from the 50 Mb kit (813 samples) to V4 kit (107 samples). As discussed later, we determined that separating samples into batches prior to filtering resulted in a higher quality variant dataset. We separated our samples into six different sample sets (see Methods) before filtering each batch in parallel (Additional file 5). For simplicity, we present data statistics for the batch containing the largest number of samples (batch 4: 688 samples).

Batch 4 contained 448,862 unfiltered SNVs (288,200 known and 160,662 novel) with 304,124,594 genotypes (272,602,882 homozygous reference and 31,521,712 non-reference genotypes; the average variant has 678 samples with a non-missing genotype) in the 688 samples. The VQSR filter removed 12.5% of these variants, with 392,826 SNVs remaining (261,570 known and 131,256 novel). At these VQSR filtered sites, 88.3% of the genotypes were retained (268,632,214 total genotypes with 242,868,311 homozygous reference and 25,763,903 non-reference genotypes; the average variant having 684 samples with a non-missing genotype). All 10 of the samples used for genotype concordance are present in batch 4. Therefore, the genotype concordance remains the same as the values presented for the entire dataset (Table 1). In contrast, the Ti/Tv calculation is now based on a smaller number of SNVs; thus, batch 4 has a different Ti/Tv ratio than the ratio presented for all 920 samples. In this batch, the unfiltered variant dataset has a Ti/Tv of 2.39 (1.93 novel and 2.71 known), while the VQSR filtered dataset has a Ti/Tv ratio of 2.63 (2.21 novel and 2.88 known) (Table 2).

### Filtering low quality genotypes improves concordance

**Figure 2:**
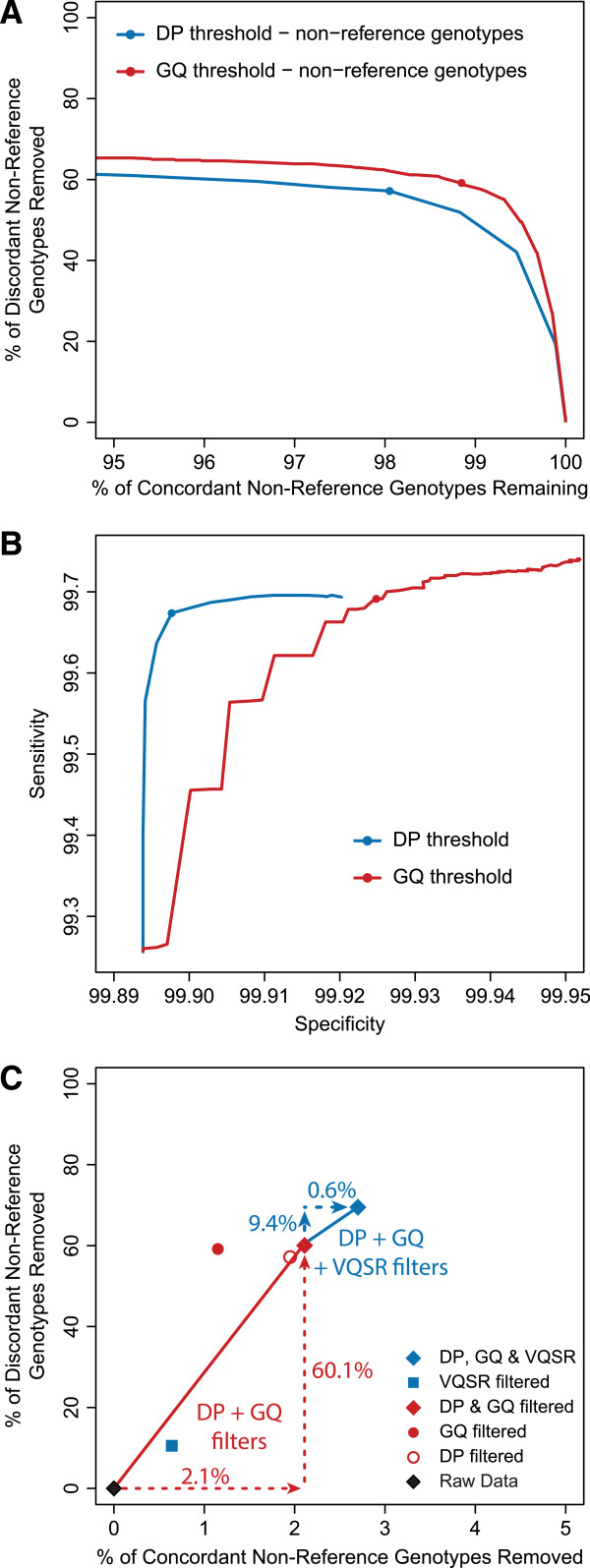
**Improved concordance**, **sensitivity and specificity of WES data using genotype filters.** Plots illustrate the non-reference concordance and sensitivity versus specificity between array and sequencing genotypes for 10 samples. **A)** The percent of non-reference discordant calls removed is plotted versus the percent of non-reference concordant calls retained at increasing quality thresholds. **B)** Sensitivity versus specificity is plotted at increasing quality thresholds. For **A)** and **B)**, blue line represents changing DP thresholds and the red line represents change GQ thresholds. Chosen filter thresholds (DP ≥ 8 and GQ ≥ 20) are indicated by points on these lines. **C)** Summarizes the effect that the chosen genotype filters (both DP and GQ) have on non-reference concordant and discordant genotype calls with and without the VQSR filter.

To evaluate how DP and GQ filters would affect concordance rates, we calculated genotype concordance at increasing DP and GQ thresholds and plotted the percent of discordant genotypes removed versus the percent of concordant retained for non-reference array genotypes (Figure 2A, Additional files 6, 7 and 8). We observed that as quality thresholds increased, genotype concordance, sensitivity, and specificity also increased before eventually reaching a plateau (Figure 2 and Additional file 6). At this plateau, increasing thresholds continued to remove variants without yielding concordance improvements. We chose a filtering threshold for each metric that was not based on this threshold, but that theoretically provided greater than 99% confidence for a genotype. For DP, we selected a minimum threshold of eight reads, corresponding to a 2 × (1/2)^8^ chance (<1%) that a biallelic variant would appear to be monoallelic by random chance, assuming a two-tailed binomial model where each allele of a biallelic variant has a 50% chance of being in each read. For GQ, we selected a minimum threshold of 20, corresponding to a Phred quality score with 99% accuracy. To see how different combinations of DP and GQ thresholds affect the genotype concordance, see Additional files 7 and 8.

After applying these genotype filters to the unfiltered data, we compared our results to the quality of unfiltered and VQSR filtered genotypes (Tables 1 and 2, Figure 2C, Additional files 1 and 3). When combined, the DP and GQ genotype filters improved the non-reference genotype concordance to 99.70% after removing 60.1% of the non-reference discordant genotypes. These filters also improve the sensitivity and specificity to 99.50% and 99.93%, respectively. When the VQSR filter is applied subsequent to the DP and GQ genotype filters, further improvement is observed, with 69.5% of the non-reference discordant genotypes removed, a concordance of 99.77%, a sensitivity of 99.62% and a specificity of 99.99% (Figure 1, Additional files 1 and 3).

Applying these DP and GQ genotype filters to the 688 samples in the batch 4 dataset removes 7.5% of the non-reference genotypes (2,361,951 of 31,521,712 non-reference genotypes and 15,564,172 of 272,602,882 reference genotypes; Additional file 5). If we extrapolate the observed concordance improvement to all the variants in all 688 samples from batch 4, we would expect to reduce the number of discordant non-reference genotypes in the entire filtered dataset by >60% (from ~233,261 to ~87,479 genotypes).

### Filtering low quality variants improves the Ti/Tv ratio

**Figure 3:**
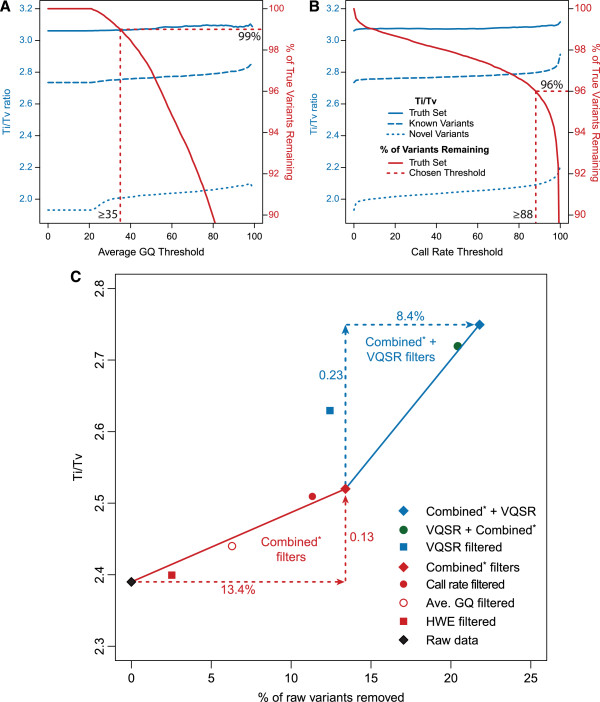
**Improved Ti/Tv ratios in WES data using variant filters.** Plots illustrate Ti/Tv improvement at different thresholds of **A)** average GQ and **B)** call rate. In these plots, Ti/Tv ratios (blue) for novel (dotted line), known (dashed line) and true (solid line) variants are juxtaposed against the drop in sensitivity (red) as the variant filtering thresholds increase. Chosen thresholds are show by the red dashed lines. **C)** The Ti/Tv improvement after each of the variant filtering steps is summarized. In addition, the result from an alternative filtering order, where VQSR is applied prior to the combined variant filters, is also displayed (green circle). *Combined filters refers to HWE, average GQ and call rate filters applied together.

To examine whether filters based on HWE, variant quality or call rate can meaningfully improve the variant data quality, we measured their effect on variant quality by examining changes in genotype concordance (Additional file 9) and in the Ti/Tv ratio at different filtering thresholds (Table 3 and Figure 3). As a proxy for variant quality, we calculated the average GQ value for each variant (sum of the individual genotype GQ values divided by the number of genotypes at a variant site).

**Table 3:** Variant filtering of WES data improves Ti/Tv ratios

**Filters**	**Variants removed**	**Number of variants (% of unfiltered)**	**Ti/Tv**				**p-value**^**a**^
			**Novel**	**Known**^**†**^	**Truth**^**‡**^	**All**	
None	0	448,862 (100%)	1.93	2.71	3.05	2.39	N/A
VQSR	56,036	392,826 (87.5%)	2.21	2.88	3.07	2.63	<10^-320^
HWE	11,855	437,007 (97.4%)	1.93	2.73	3.06	2.40	1.42 × 10^-21^
Ave. GQ	33,083	415,779 (92.6%)	2.00	2.73	3.06	2.47	1.13 × 10^-265^
Call Rate	51,117	397,745 (88.6%)	2.09	2.78	3.08	2.51	<10^-320^
Combined^*^	59,952	388,910 (86.6%)	2.09	2.80	3.09	2.52	<10^-320^
Combined^*^ + VQSR	97,840	351,022 (78.2%)	2.38	2.96	3.10	2.75	<10^-320^ (3.72 × 10^-106^)^b^
VQSR + Combined^*^	92,091	356,771 (79.5%)	2.34	2.94	3.10	2.72	<10^-320^

^†^Variants found in NCBI dbSNP Build 135.

^‡^Variants found in HapMap phase 3 release 3.

^*^Combination of HWE, Ave. GQ and Call Rate filters.

^a^p-value based on a hypergeometric test of whether the removed variants were enriched for Tv over Ti vs. the unfiltered variant sets.

^b^p-value based on a hypergeometric test of whether the variants that differed between Combined + VQSR variant sets and VQSR + Combined variant sets were enriched for Tv over Ti.

First, we filtered out 11,855 variants (2.6% of the original variants) that significantly deviated from HWE (p ≤ 0.05 after Bonferonni correction). Since such a small number of variants are removed, we only observe a slight increase in the Ti/Tv ratio (from 2.39 to 2.40; Table 3). This improvement is due to a significant enrichment in the filtering for the removal of Tv variants over Ti variants (P = 1.42x10^-21^; Table 3 and Additional file 10). In addition, we see a slight improvement in non-reference concordance (0.02% improvement before applying the VQSR filter; Additional file 9). The HWE filter removes more FPs (299 of 446 before VQSR filtering and 11 of 36 after VQSR filtering) than FNs (22 of 220 before VQSR filtering and 7 of 168 after VQSR filtering; Additional file 9). Overall, we observed a slight improvement in Ti/Tv, concordance, sensitivity and specificity following HWE filtering that suggests that this generally standard quality filter may be applicable to sequencing projects that will be tested for association.

We next calculated Ti/Tv ratios at different filtering thresholds to determine whether average GQ (Figure 3A) or call rate (Figure 3B) filters can improve variant quality. We contrasted the Ti/Tv improvement against the sensitivity for detecting “true” variants (variants found in HapMap phase 3 release 3; the same dataset utilized by VQSR to establish sensitivity tranches in GATK best practices). In addition, we also separated known from novel variants. For average GQ (Figure 3A), improvement begins at a threshold of 20 due to the fact that we previously removed all genotypes with GQ < 20. Following this, the Ti/Tv then quickly increased, most notably in the novel variants, before reaching a plateau. In addition, as we increased the average GQ threshold, the number of true variants remaining dropped quickly. As with the VQSR filter, we chose a sensitivity threshold of 99%, which corresponded to variants with an average GQ ≥35. This captured the majority of the Ti/Tv increase while sacrificing only a minimal percentage of the true variants in the dataset. In total, the average GQ filter improved the overall Ti/Tv by 0.08 (2.39 to 2.47) while only removing 7.4% of the original unfiltered variants (Table 3). Again, while this is only a slight improvement in Ti/Tv, the filter is significantly biased towards the removal of Tv variants (P = 1.13x10^-265^; Table 3 and Additional file 10). There is also a slight concurrent improvement in sensitivity and specificity (17 FPs and 1 FN removed; Additional file 9) that additionally suggests this filter is advantageous when applied to this dataset.

As the call rate threshold was raised (Figure 3B), we observed a gradual increase in Ti/Tv. This is accompanied by a gradual drop in the number of true variants until very high call rate thresholds are reached, where the number of true variants dropped rapidly. To avoid this rapid drop while maximizing the gain of Ti/Tv, we chose to preserve a true variant threshold of 96%, which corresponded to variants with call rates ≥88%. Again, this significantly improved the overall Ti/Tv (from 2.39 to 2.51, P < 10^-320^), while only removing 11.4% of the overall unfiltered variants (Table 3 and Additional file 10). In addition, this filter improved concordance by 0.02% while removing an additional 42 FPs and 13 FNs (Additional file 9).

We observed that using a combination of HWE, average GQ and call rate variant filters provided a significant increase in Ti/Tv (2.39 to 2.52) while removing 13.4% of the unfiltered variants. Importantly, when the VQSR filter is applied subsequent to these three variant filters, we saw the greatest improvement of Ti/Tv (2.75) with a concomitant loss of 21.8% of the variants (Figure 1, Figure 3C and Table 3).

### Order of filtering steps is important

We next determined the optimal order of implementing our variant and VQSR filters to obtain the highest quality variant dataset. We compared the above order, which applied VQSR filtering subsequent to our variant filters, to an alternative filtering order, with VQSR filtering applied before our variant filters. In this alternative order, fewer variants were removed (20.5% versus 21.8%), but the resulting Ti/Tv was lower (2.72 versus 2.75; Table 3 and Figure 3C). To determine if this order consistently improved the Ti/Tv ratios of the filtered variants, we also compared the results from the different orders of filtering on each of the other five batches (Additional file 11). In each case, applying the VQSR filter after performing the manual variant filters consistently resulted in a higher filtered Ti/Tv ratio. In addition, we tested whether the extra variants removed by this filtering order were enriched for Tv variants (Additional file 11). Again, in each case the extra variants removed by performing VQSR filtering after the manual variant filters were significantly enriched for Tv variants. Therefore, applying VQSR filtering as the final step in our method provided the highest quality variant dataset.

### Batch effects cause data heterogeneity in large-scale exome sequencing projects

Variant and genotype quality scores can differ depending on the chemistry and sequencing protocols used to generate the data and will frequently result in batch effects if these factors are not taken into account. To investigate the effect that splitting the data into batches had on the final variant dataset, we performed our quality control steps with (“batched”) and without (“unbatched”) partitioning the samples based on differences in their processing (Table 4 and Additional file 5).

**Table 4:** Splitting samples by batch (“batched”) retains more high quality variants

	HWE^*^	**Call rate**^**†**^	**Ave.GQ**^**‡**^	**VQSR**^**¥**^	**Total**
Number of variants filtered from “unbatched” dataset	14,209	197,540	0	26,967	238,716
Number of filtered variants found in “batched”	1,983	135,031	N/A	2,050	139,064
Ti/Tv of filtered variants found in “batched”	2.64	2.20	N/A	2.16	2.20

^*^Used – hwe in vcftools to remove variants with Bonferroni-corrected p-value < 0.05.

^†^Used – geno in vcftools to remove variants with call rates < 88%.

^‡^Used an awk command to remove variants with average GQ < 35.

^¥^Filtered VQSR processed variants at the 99% sensitivity tranche.

After filtering, the unbatched dataset contained 334,358 variants (227,202 known and 107,156 novel) (Table 4). Since the target definitions changed between the 50 Mb and V4 capture kits, some variants are “off target” in one kit and are “on target” in the other. This can lead to low quality variants being retained in the unbatched filtered dataset, even though they would be considered “off-target” in a subset of the batched data and appropriately removed. We identified 2,304 such variants in the unbatched filtered dataset.

The batched dataset contained 471,118 variants (311,475 known and 159,643 novel) (Table 4). Of these variants, 139,064 were not found in the unbatched dataset. The vast majority (97.1%) of these batched-specific variants were filtered out of the unbatched dataset during the call rate filtration step. These 139,064 variants had low call rates in some batches, but a high call rate (≥88%) in at least one batch. This call rate heterogeneity between batches was primarily due to the use of different target definitions in the two capture kits, but could also be caused by any factor that affects depth of coverage in batches.

We determined the quality of the variants unique to each dataset by measuring both their genotype concordance and their Ti/Tv ratio. The variants unique to the unbatched dataset were found to have a non-reference genotype concordance of 91.39% (138 of 151 non-reference genotypes from 111 variants intersecting the array data; 853 of 857 concordant reference genotypes, or 99.53%), while the non-reference genotype concordance of the variants unique to the batched dataset was much higher at 98.81% (1326 of 1342 non-reference genotypes from 4,984 variants intersecting the array data; 9462 of 9462 concordant reference genotypes, or 100%). In addition, variants unique to the batched dataset had a higher Ti/Tv ratio than the variants unique to the unbatched dataset (2.20 versus 1.67). Overall, we determined that by batching samples prior to performing filtering, we retained 40.9% more high quality variants emphasizing the importance of accounting for target and chemistry variation during variant and genotype filtration.

### Imputation of common SNPs

Imputation methods utilize sequenced variants from within the exome to calculate genotype likelihoods at positions outside of the exome. We obtained imputed genotypes at 9,711,915 variant sites in all 920 samples using a combination of GATK and Beagle (see Methods). However, since these imputed sites have little to no sequencing coverage, it is difficult to assess the accuracy and quality of the resulting data. Therefore, we again took advantage of the HumanExome array by calculating imputed genotype concordance using 390,958 high quality (GCScore ≥ 0.3) array genotypes (238,343 homozygous reference and 152,615 non-reference) from 10 of the samples.

**Figure 4:**
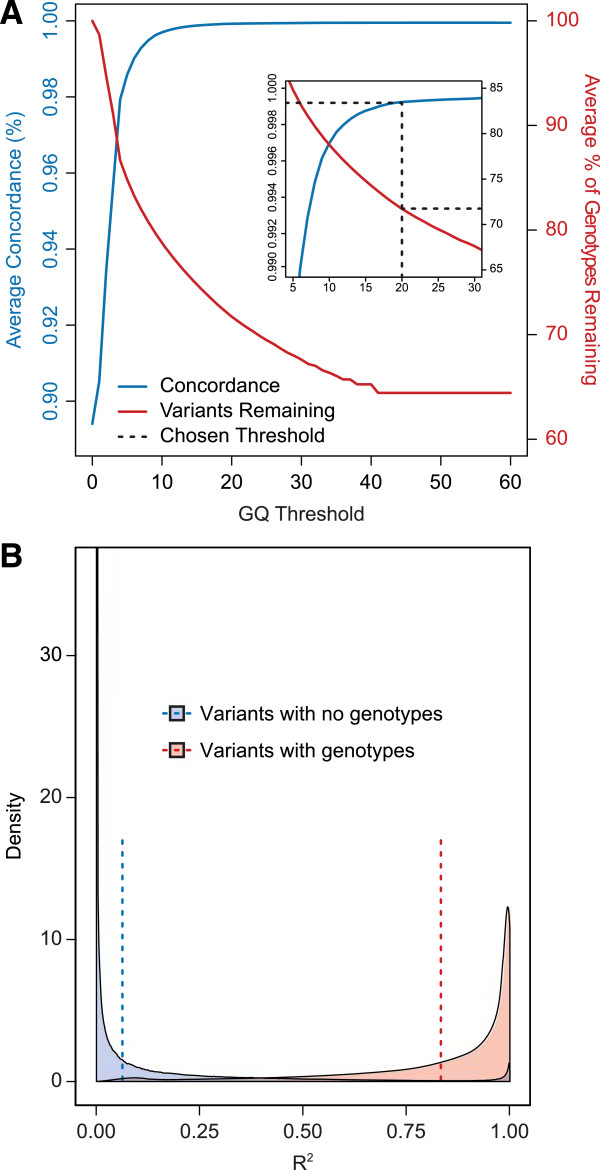
**Applying a GQ filter improves the quality of imputation results from WES data.** Plots illustrate data quality improvement seen after applying GQ threshold. **A)** Plots the average concordance (blue line) improvement between array and sequencing genotypes for 10 samples as the GQ threshold increases. Coupled with this concordance improvement is the average percent of genotypes that remain (red line) with GQ values above that threshold. **B)** At the GQ > 20 threshold, this plot shows that variants removed (blue) due to loss of all genotypes have generally lower quality (as measured by R^2^) compared to variants containing at least one genotype (red). Mean values for each distribution are shown by the dotted lines.

Much like sequencing data, each imputed genotype is given a corresponding GQ value, allowing us to assess genotype quality at various GQ thresholds (Figure 4). We observe that as the GQ threshold increased, the non-reference concordance with the array genotypes increased with a concomitant drop in the number of genotypes remaining. To achieve a 99% confidence in the genotype calls, we again set the threshold at GQ ≥ 20 (Figure 4A). This removed almost all of the discordant genotypes (31,452 of 31,619 non-reference discordant genotypes, or 99.5%; 9,765 of 9,811 reference discordant genotypes, or 99.5%) and significantly improved the concordance (non-reference: 79.3% to 99.8%; reference: 95.9% to 99.98%). However, unlike with the genotypes obtained from sequencing, this removed a much larger proportion of the genotypes (45.4% of the non-reference genotypes and 17.3% of the reference genotypes), suggesting that the unfiltered genotypes from imputation contain more low quality genotypes than the unfiltered genotypes from sequencing.

We next filtered the dataset to remove non-informative variants created by the GQ filtering step. These included “monoallelic” variants, where all unfiltered genotypes are homozygous for the same allele, and “no genotype” where all genotypes at a variant site were removed by the GQ filter. From the imputed dataset, 2,625,290 (27.0%) of the variants were “monoallelic” and 4,680,753 (48.2%) were “no genotype” variants after applying the GQ filter. These “no genotype” variants were imputed with low likelihoods, suggesting they were of poor quality. This was confirmed by assessing the R^2^ distribution for these variants (Figure 4B). Variants with no genotypes passing the GQ threshold generally had a lower R^2^ value than variants with genotypes passing the filter (average 0.06 vs 0.83). After removing all “monoallelic” and “no genotype” variants, we retained 2,405,872 imputed variants (24.8% of the unfiltered data) with 1,371,079,415 high quality genotypes (954,090,448 homozygous reference and 416,988,967 non-reference genotypes; the average variant has 859 samples with a non-missing genotype).

Lastly, we compared the improvement in imputed data quality using the GQ filter to using a simple R^2^ cutoff. Many genome-wide association studies use a hard cutoff of R^2^ > 0.3 to filter imputed data [34]. However, this R^2^ filter removed fewer discordant genotypes (25,805 of 31,619, or 81.6%, of non-reference discordant genotypes and 8,110 of 9,811, or 82.7%, of reference discordant genotypes) and resulted in a lower concordance (94.7% non-reference concordance and 99.2% reference concordance) than using the GQ filter. Therefore, the quality improvement observed using the GQ filter is superior to using a R^2^ > 0.3 cutoff.

## Discussion

We developed filters at both the genotype and variant levels (Figure 1). For genotypes, we selected thresholds for DP (≥8 reads) and GQ (≥20) to filter out genotypes with <99% likelihood. We demonstrated that these thresholds improve genotype quality by assessing the improvement in genotype concordance with high quality array genotypes. Both thresholds individually improved genotype concordance, with greater improvement when combined (Figure 2C and Tables 1 and 2). Since these genotype quality thresholds were chosen to optimize genotype probability (based on the quality metric and independent of the actual data), these values can be applied universally to filter sequencing datasets. While some researchers may prefer higher specificities (coupled with a decreased sensitivity), the genotype concordance and sensitivity versus specificity curves (Figures 2A and 2B) suggest that more stringent thresholds may provide only very minor quality improvements that are outweighed by a significant loss of genotypes. For example, selecting for genotype likelihoods greater than 99.9% (instead of 99%) would require thresholds of DP ≥ 11 and GQ ≥ 30, but would only increase overall concordance by 0.0018% while removing an additional 1.14% of all genotypes. Therefore, to achieve a 99% genotype likelihood, we recommend thresholds of DP ≥ 8 and GQ ≥ 20 be chosen when this filtering method is applied to sequencing studies.

We found that the DP and GQ filters made more of an impact on non-reference calls than reference. The VQSR filter was adept at removing FPs, which were primarily non-reference calls at reference sites. However, it performed worse when asked to remove reference calls at non-reference sites, removing only 10.5% of FN calls. By including the DP and GQ filters, the FN calls were reduced by 69.5%. We also observed that non-reference genotypes were preferentially affected in the imputed data. For non-reference genotypes, the concordance was initially poor (79.3%) and was improved to 99.8% with a GQ filter. However, the same GQ filter only increased the reference concordance from 95.9% to 99.98%. These increases are relevant for rare variant association tests as they rely on high accuracy at non-variant sites.

At the variant level, while we chose a universal threshold for HWE (Bonferroni-corrected P ≤ 0.05), we empirically determined thresholds for average GQ (≥35) and call rate (≥88%). Of these three filters, the most crucial is the call rate filter, since it provided the largest quality improvement (Ti/Tv increase from 2.39 to 2.51; Table 3). While HWE and average GQ have less significant Ti/Tv improvements, this is partially due to the smaller number of variants that are removed by these filters. While these two filters have a smaller effect on the Ti/Tv ratio, both filters remove a significantly larger proportion of Tv variants than would be expected by chance (Table 3 and Additional file 10) and also improve the concordance (Additional file 9). This suggests that they are both beneficial to this filtering method. Since these thresholds were empirically chosen to optimize Ti/Tv while minimizing the loss of “true” variants, researchers may prefer to similarly determine these thresholds for their own datasets, rather than relying on these specific thresholds. Therefore, unlike the genotype filter thresholds, the variant filters should not be universally applied, but can be empirically determined using the methods that we have demonstrated.

We demonstrate the importance of grouping samples into batches according to technical methodologies prior to filtering for producing high quality variants without sacrificing sensitivity. Differences in sequence depth coverage between batches can lead to significant call rate differences. Since we recommended a call rate filter as part of our method, these differences can lead to the removal of variants with sufficient call rates in one batch even if other batch call rates fall below the filtering threshold. To illustrate the importance of separating samples into batches, we demonstrated that 139,064 high quality variant (with 98.81% non-reference genotype concordance, 100% reference genotype concordance, and 2.20 Ti/Tv ratio; Table 4) were lost from the “batched” variant set when all 920 samples were filtered together in an “unbatched” manner. Of these variants, 97.1% were removed by the call rate filter due to differences in call rates between batches caused by coverage heterogeneity. Based on these results, separating batches prior to filtering, then recombining variants before performing downstream analyses is highly recommended.

We also demonstrated that the order of filtering has a significant effect on the quality of the final variant dataset. When VQSR is applied before our suggested filters, the Ti/Tv was lower than when the same thresholds are applied before running VQSR (Table 3). However, coupled with this higher Ti/Tv (and implied increase in quality) was the loss of an additional 1.3% of the unfiltered variants. Since many downstream analyses, especially burden and collapsing analyses, benefit most from a highly specific dataset with low levels of noise, we recommend running our suggested filters prior to performing VQSR filtering.

Lastly, we showed that by filtering imputed genotypes we significantly improved the concordance of the data. In the same way as genotypes generated from sequencing data were filtered, we selected a threshold that provides a genotype likelihood greater than 99% by filtering for GQ ≥ 20. This resulted in a compromise between an increased accuracy (ie: higher genotype concordance) and a minimized loss of genotypes (Figure 4) and can be applied universally to any imputed dataset.

The methods described provide the highest utility for rare variant association analyses. While genotyping errors reduce the statistical power for common variants, this decrease is more pronounced for variants with low MAF. Therefore, rare variant association tests, which collapse multiple variants with low MAFs, are particularly sensitive to genotyping errors and should benefit the most from the described robust filtering methods. Therefore, although large numbers of genotypes are removed during filtering to improve the quality of the dataset, the overall power to detect significance should increase by removing these errors from the downstream rare variant association analyses.

## Conclusion

By utilizing the described processing and filtering method, we were able to improve: 1) the quality of the genotypes - 99.77% non-reference concordance in the filtered dataset versus 99.26% non-reference concordance in the unfiltered genotypes and 99.33% non-reference concordanance after VQSR filtering alone; 2) the Ti/Tv ratio of the final variants - 2.75 in the filtered dataset versus 2.39 in the unfiltered dataset and 2.63 in VQSR filtered dataset; and 3) the number of variants identified - 471,118 “batched” variants versus 334,358 “unbatched” variants. In addition, we improved the quality of genotypes from imputation - 99.8% non-reference concordance in the filtered genotypes versus 79.3% non-reference concordance in the unfiltered genotypes.

Our results demonstrate effective methods for improving the quality of WES data using easily implemented and publically available tools. These methods are applicable to sequencing studies that identify germline variants, but are not suitable for somatic mutation detection. Additionally, these filters can be applied to studies that have less than 30 samples which cannot optimally utilize GATK VQSR filtering. In these studies, the genotype and variant filters described may have increased utility since VQSR filtering may not sufficiently improve the variant quality of the dataset.

Overall, the methods described represent significant improvements over the standard practices for sequencing data processing to decrease the number of errors carried forward into association, burden and collapsing analyses conducted in studies of complex diseases.

## Methods

### Sequencing and variant calling

The Tromsø Study is a single center prospective follow-up study with repeated health surveys of inhabitants in the municipality of Tromsø, Norway [40]. We sequenced the exomes of 920 individuals from the fourth survey of the Tromsø Study (Tromsø IV) conducted in 1994-95 [40]. DNA was isolated from whole blood and stored at -70°C at the national CONOR biobank, located at the HUNT Biobank in Levanger, Norway. Agilent SureSelect 50 Mb or V4 capture kits (813 and 107 samples, respectively) were used to target exome regions (>21,000 genes and >500 miRNA) from genomic DNA. Samples were then multiplexed and sequenced on an Illumina HiSeq 2000, with density clusters generated using either the Illumina TruSeq PE cluster kit v2-cBot-HS or v3-cBot-HS (93 and 827 samples, respectively).

Paired-end 100 bp sequenced reads were mapped to the human genome (hg19 with unmapped and mitochondrial chromosomes removed) using BWA [41] with default parameters for paired end alignment. Reads were then processed (duplicates removed, reads realigned around indels, and quality scores recalibrated) and variants called using a combination of Picard and GATK (software version 1.6 and best practices v3) [24]. For the “VQSR filter”, variant quality scores were recalibrated using VQSR and filtered at the recommended 99% sensitivity tranche.

### Human exome beadchip assay

Ten of the 920 samples were analyzed using the Illumina Infinium HD HumanExome BeadChip Assay. Samples were processed according the manufacturer’s specifications. Genotypes were called using GenomeStudio (v2011.1) using default cluster positions and filtered for GenCall Score ≥ 0.30. Genotypes were converted from Illumina TOP orientation to genome orientation (hg19) using the HumanExome-12v1_A files generated through the Wellcome Trust Center for Human Genetics (http://www.well.ox.ac.uk/~wrayner/strand/). Sites reported as “Cautious Sites” (http://genome.sph.umich.edu/wiki/Exome_Chip_Design#Cautious_Sites) were removed.

### Separating samples into six batches

We grouped the 920 samples into six different batches to account for the different target capture versions, sequencing reagents, and sample DNA input quantities used during the project’s sequencing phase (Additional file 12). Batch 1 consists of 93 samples with sequencing data generated using the TruSeq PE cluster kit v2 and the Agilent SureSelect 50 Mb capture kit. Next, 720 samples were sequenced using the improved TruSeq PE cluster kit v3 and the Agilent SureSelect 50 Mb capture kit. These samples were split into three batches: 25 samples with low input DNA (500 ng) (batch 2), seven samples with low coverage that required resequencing (batch 3), and the 688 remaining samples (batch 4). Finally, 107 samples were sequenced using both the improved TruSeq PE cluster kit v3 and the improved Agilent SureSelect V4 capture kit. These samples were split into two batches based on input DNA: seven with whole genome amplified DNA (batch 5) and 100 with genomic DNA (batch 6).

### Imputation at common SNPs

WES data was used to impute additional genotypes using haplotypes from the European samples of the 1000 Genomes Project (301 unrelated individuals) following the previously published methodology [42]. In this method, 9,711,915 common sites (allele frequency > 0.005 and < 1 in the European individuals) were analyzed using GATK Unified Genotyper to generate genotypes and genotype likelihoods from WES aligned reads. These genotype likelihoods were then used as input for Beagle [43], which recalculates the probability and determines the most likely genotype at each site in each sample.

### Genotype and variant filters

Genotype (both from sequencing and imputation) and variant filters were applied using vcftools [44]. For genotypes, the “minGQ” and “minDP” options were used to filter genotype quality and depth, respectively. For variant filtration, the “hwe” option was used to filter variants that deviated from HWE, while the “geno” option was used to filter variants by call rate. A simple AWK script was created to calculate and filter based on average genotype quality (available upon request).

## Abbreviations

WES: Whole exome sequencing; GATK: Genome Analysis Toolkit; MAF: Minor allele frequency; SNV: Single nucleotide variant; Ti/Tv: Transition to transversion ratio; DP: Depth of sequencing data; GQ: Genotype quality; HWE: Hardy-Weinberg equilibrium; TN: True negative; TP: True positive; FN: False negative; FP: False positive.

## Competing interests

The authors declare that they have no competing interests.

## Authors’ contributions

ARC carried out the filtering methods and analysis of the genotype and variant quality. ENS participated in design of the study, interpretation of the results, and writing of the manuscript. HK created and ran the bioinformatics pipeline that generated the variant calls from the sequencing read data. SB was involved in sample selection and project design. KJ oversaw the sample processing, sample sequencing and data generation. JBH oversaw the sample selection and co-led project design. KAF co-led the project design, oversaw the project and participated in writing the manuscript. All authors have read and approved the final manuscript.

## Supplementary Material

Additional file 1 Table WES genotype concordance in 10 samples after VQSR and genotype filters, including number of genotypes and reference and non-reference concordance. Click here for file

Additional file 2 Figure Depth and quality of genotypes remaining in the VQSR filtered dataset. Click here for file

Additional file 3 Table WES sensitivity and specificity compared to exome array genotypes in 10 samples after VQSR and genotype filters. Click here for file

Additional file 4 Figure Average target coverage of 920 sequence samples shows batch effects. Click here for file

Additional file 5 Figure Detailed view of batched versus unbatched filtering. Click here for file

Additional file 6 Figure Comparison of concordance, specificity, and sensitivity at ranges of DP and GQ filters before and after VQSR filtering. Click here for file

Additional file 7 **Table Concordance between reference (7a) and non-reference (7b) array genotypes and WES genotypes for different combinations of DP and GQ filters.** Tables show A) concordance from “raw”, or unfiltered, genotypes before VQSR; B) concordance from VQSR filtered genotypes. Click here for file

Additional file 8 Table Sensitivity and specificity estimates of WES calls for exome array genotypes at different DP and GQ thresholds. Click here for file

Additional file 9 Table WES genotype concordance and sensitivity versus specificity compared to exome array genotypes in 10 samples after VQSR and variant filters. Click here for file

Additional file 10 Table Using a hypergeometric test to look for statistical significant enrichment in the filtering of Tv over Ti variants by each variant filter. Click here for file

Additional file 11 Table The effect of the order of filtering on Ti/Tv ratios in all six batches. Click here for file

Additional file 12 Table Samples are sorted into six batches. Click here for file
