# Supplementary material for: Effective filtering strategies to improve data quality from population-based whole exome sequencing studies
Source: BMC Bioinformatics. 2014 May 2;15:125. doi: 10.1186/1471-2105-15-125 (PMC4098776; doi:10.1186/1471-2105-15-125)
Supplement: Additional file 2 — Figure Depth and quality of genotypes remaining in the VQSR filtered dataset. [file 1471-2105-15-125-S2.pdf]

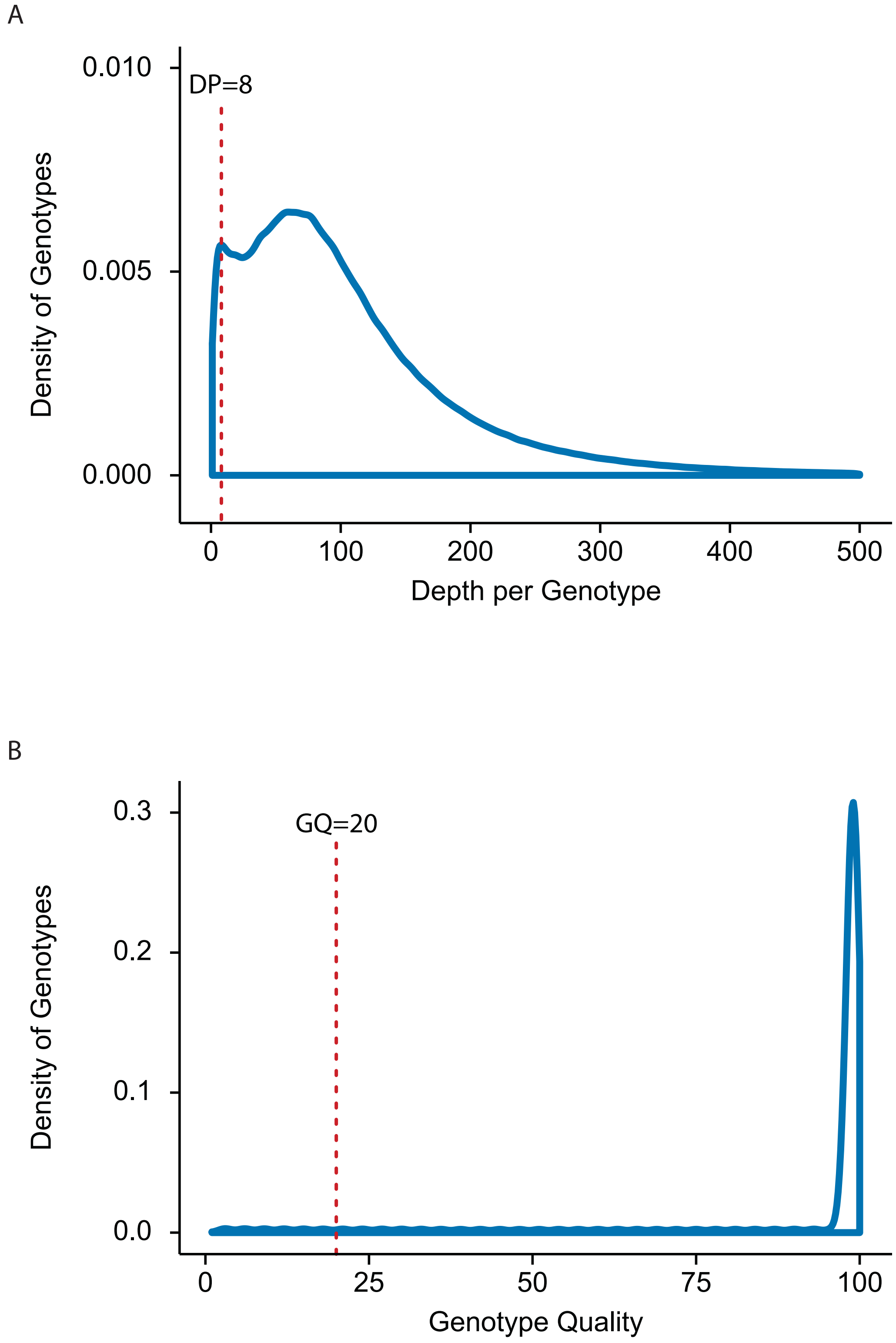

Additional File 2: Depth and quality of genotypes remaining in the VQSR filtered dataset.
A) Depth (DP) of genotypes found in the VQSR filtered dataset. B) Quality (GQ) of genotypes found in the VQSR filtered dataset. For both plots, the red dotted lines shows the utilized thresholds in the filtering methods.
