# Supplementary material for: Effective filtering strategies to improve data quality from population-based whole exome sequencing studies
Source: BMC Bioinformatics. 2014 May 2;15:125. doi: 10.1186/1471-2105-15-125 (PMC4098776; doi:10.1186/1471-2105-15-125)
Supplement: Additional file 4 — Figure Average target coverage of 920 sequence samples shows batch effects. [file 1471-2105-15-125-S4.pdf]

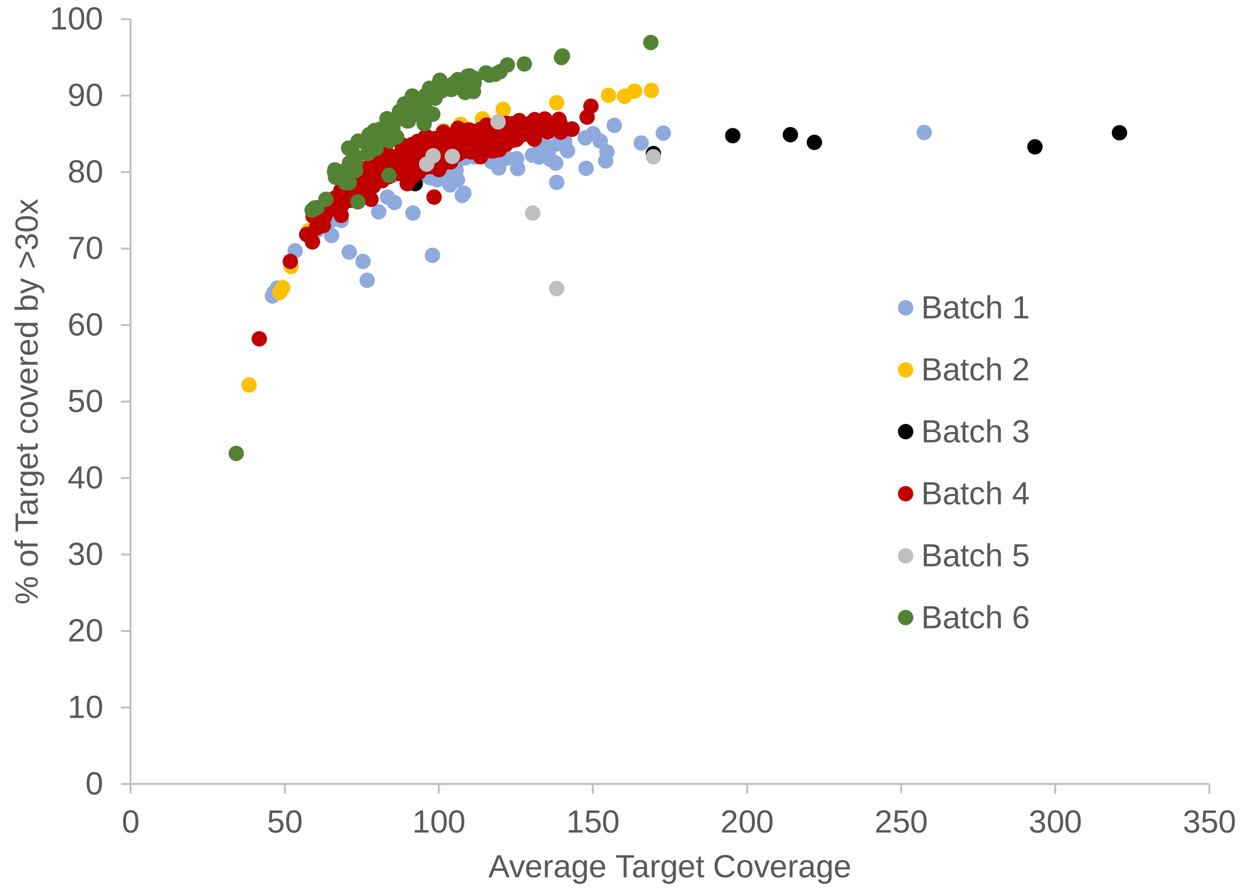

Additional File 4: Average target coverage of 920 sequence samples shows batch effects

The plot shows the average target depth of coverage versus the percent of the target covered by more than 30 reads for each sample from the six batches. Noticeable batch effects can be seen. Batch 4 (red) is the batch discussed throughout the manuscript before all batches are merged into a final filtered variant dataset.
