# Supplementary material for: Effective filtering strategies to improve data quality from population-based whole exome sequencing studies
Source: BMC Bioinformatics. 2014 May 2;15:125. doi: 10.1186/1471-2105-15-125 (PMC4098776; doi:10.1186/1471-2105-15-125)
Supplement: Additional file 5 — Figure Detailed view of batched versus unbatched filtering. [file 1471-2105-15-125-S5.pdf]

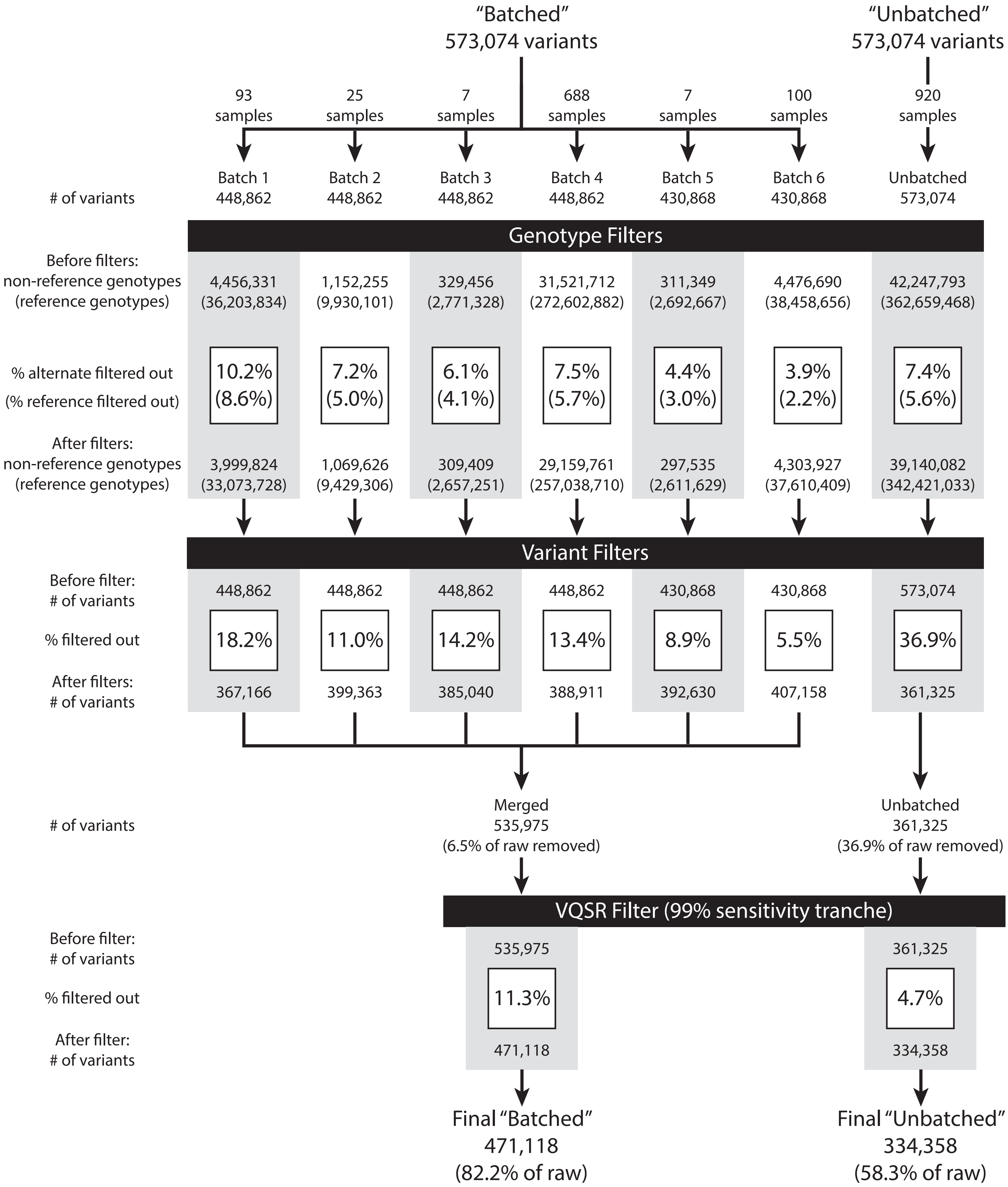

Additional File 5: Detailed view of batched versus unbatched filtering.
This schematic displays the details for filtering data in batches (left) versus filtering all 920 samples together (right). At each step, the number of variants or genotypes are shown.
