# Supplementary material for: Effective filtering strategies to improve data quality from population-based whole exome sequencing studies
Source: BMC Bioinformatics. 2014 May 2;15:125. doi: 10.1186/1471-2105-15-125 (PMC4098776; doi:10.1186/1471-2105-15-125)
Supplement: Additional file 6 — Figure Comparison of concordance, specificity, and sensitivity at ranges of DP and GQ filters before and after VQSR filtering. [file 1471-2105-15-125-S6.pdf]

A) Concordance between non-reference array genotypes and WES genotypes

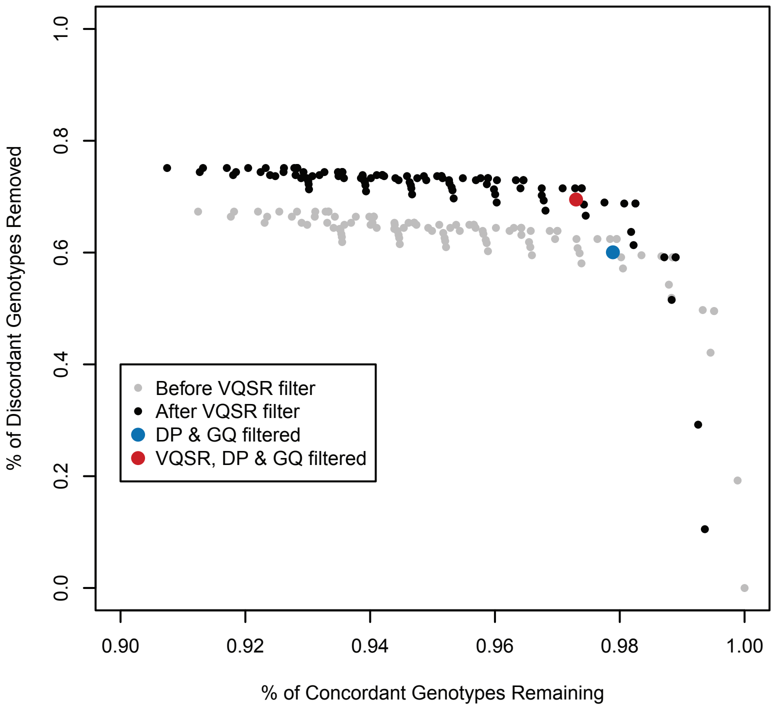

B) Concordance between reference array genotypes and WES genotypes

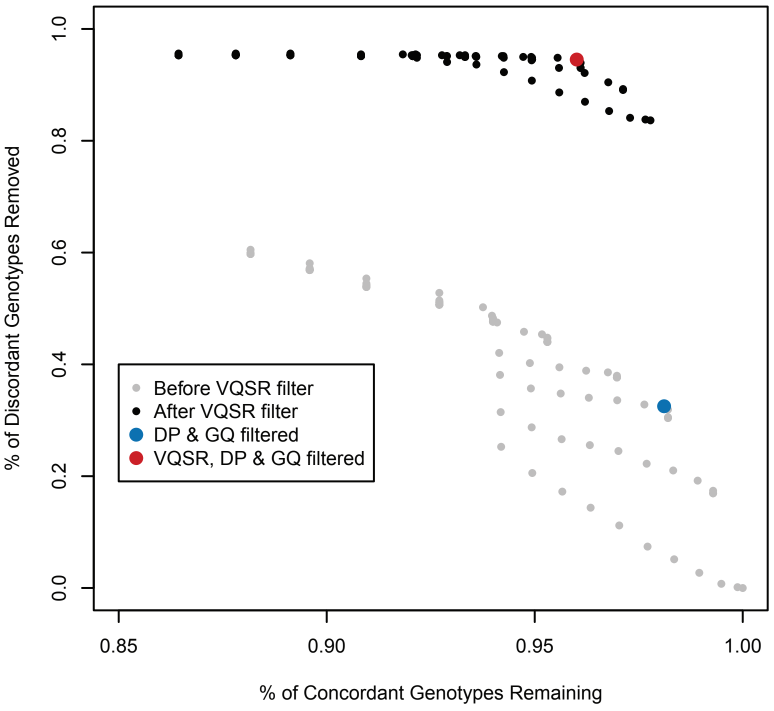

C) Sensitivity vs Specificity using all genotypes from exome arrays

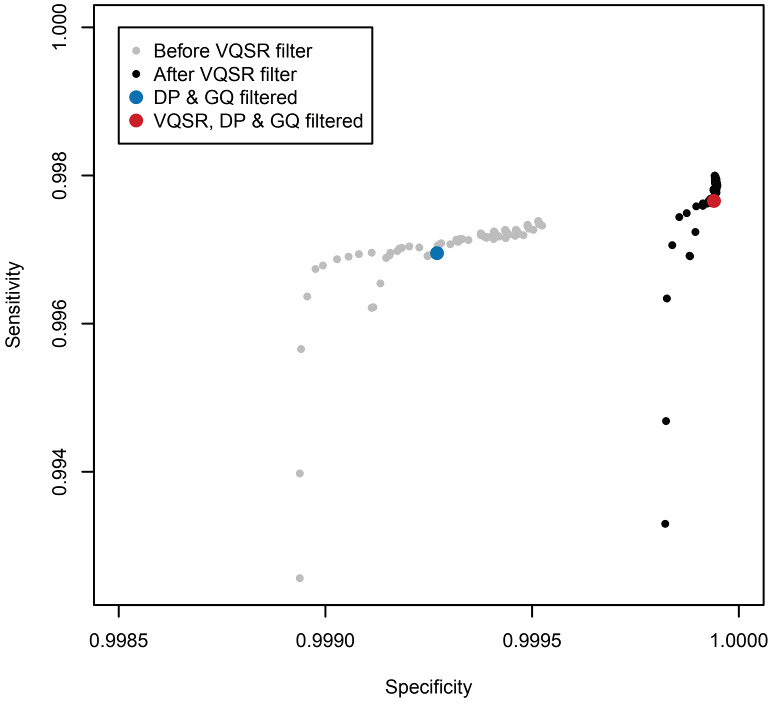

Additional File 6: Comparison of concordance, specificity, and sensitivity at ranges of DP and GQ filters before (grey circles) and after (black circles) VQSR filtering. Blue (before VQSR) and red (after VQSR) circles represent the chosen filters of DP≥8 and GQ≥20.
