# Supplementary material for: Effective filtering strategies to improve data quality from population-based whole exome sequencing studies
Source: BMC Bioinformatics. 2014 May 2;15:125. doi: 10.1186/1471-2105-15-125 (PMC4098776; doi:10.1186/1471-2105-15-125)
Supplement: Additional file 12 — Table Samples are sorted into six batches. [file 1471-2105-15-125-S12.docx]

Additional File 12. Samples are sorted into six batches

|  |  | Agilent SureSelect  target capture kit | | | |
| --- | --- | --- | --- | --- | --- |
|  |  | 50Mb | | V4 | |
| Illumina TruSeq  PE cluster kit | v2 | **1)** Standard protocol | 93 samples | 0 samples | |
|  | v3 | **2)** Low input^†^  **3)** Resequenced  **4)** Standard protocol | 25 samples  7 samples  688 samples | **5)** WGA^‡^  **6)** Standard protocol | 7 samples  100 samples |

Batch numbers are indicated by bold font.

^†^ Input DNA < 1μg

^‡^ Whole genome amplified DNA
